# The effect of aging-associated impaired mitochondrial status on kainate-evoked hippocampal gamma oscillations

**DOI:** 10.1016/j.neurobiolaging.2012.01.001

**Published:** 2012-11

**Authors:** Cheng B. Lu, Martin Vreugdenhil, Emil C. Toescu

**Affiliations:** School of Clinical and Experimental Medicine, College of Medical and Dental Sciences, University of Birmingham, Birmingham, UK

**Keywords:** Gamma oscillations, Kainate, Mitochondrial depolarization, Calcium signal, Mitochondrial protonophore

## Abstract

Oscillations in hippocampal neuronal networks in the gamma frequency band have been implicated in various cognitive tasks and we showed previously that aging reduces the power of such oscillations. Here, using submerged hippocampal slices allowing simultaneous electrophysiological recordings and imaging, we studied the correlation between the kainate-evoked gamma oscillation and mitochondrial activity, as monitored by rhodamine 123. We show that the initiation of kainate-evoked gamma oscillations induces mitochondrial depolarization, indicating a metabolic response. Aging had an opposite effect on these parameters: while depressing the gamma oscillation strength, it increases mitochondrial depolarization. Also, in the aged neurons, kainate induced significantly larger Ca^2+^ signals. In younger slices, acute mitochondrial depolarization induced by low concentrations of mitochondrial protonophores strongly, but reversibly, inhibits gamma oscillations. These data indicating that the complex network activity required by the maintenance of gamma activity is susceptible to changes and modulations in mitochondrial status.

## 1 Introduction

Normal brain aging is characterized by a mild decline in cognitive performance (Hedden and Gabrieli, 2004), that includes modules dependent on hippocampal function, as shown both in humans and animals (Erickson and Barnes, 2003). In contrast to Alzheimer's disease, principal cell loss is absent in the aged brain (Rasmussen et al., 1996), and the functional decline is attributed to more subtle functional changes at synaptic and network level (Toescu and Verkhratsky, 2007).

It has been proposed that synchronous neuronal activity in the gamma frequency band (30–80 Hz) is important for memory functions in the hippocampus by providing the millisecond precision time frame required for synaptic integration (Bartos et al., 2007; Fries et al., 2007; Lisman, 1999). Gamma oscillations have been implicated in explorative behavior and navigation (Bragin et al., 1995; Buzsáki et al., 2003; Csicsvari et al., 2003). Furthermore, changes in hippocampal gamma strength are directly correlated with activity and behavioral performance in rodents (Lu et al., 2011b; Ma and Leung, 2000) and humans (Uchida et al., 2000). Consistent with these observations, we have shown that in vitro hippocampal gamma activity, recorded in area CA3, which drives the intrinsic hippocampal gamma activity (Csicsvari et al., 2003) and evoked, in vitro, either by carbachol or kainate, was strongly reduced in aged mice, without changes in underlying synaptic connections (Vreugdenhil and Toescu, 2005). Gamma synchronization is due to the activity of a network of synaptically coupled interneurons that, while being driven by the pyramidal cell firing target the principal cells and control their firing through a fast gamma-aminobutyric acid (GABA_A_) receptor-mediated inhibition (Bartos et al., 2007; Csicsvari et al., 2003; Fisahn et al., 1998). While the pyramidal cells fire during the gamma activity at 1–3 Hz, the fast-spiking interneurons fire action potentials up to 40 Hz in a phase-locked manner (Hájos et al., 2004). Such rates of firing, particularly sustained over longer periods of time during the neurotransmitter-evoked gamma activity, are likely to impose significant metabolic demands (Huchzermeyer et al., 2008; Lu et al., 2011a). Neuronal aging is characterized, from a metabolic standpoint, by a reduction in the homeostatic reserve, defined as the neuronal capacity to respond effectively to metabolic stressors (Toescu, 2005; Toescu and Vreugdenhil, 2010), and underpinned by mitochondrial dysfunction (Kann and Kovács, 2007; Xiong et al., 2002) and alterations in Ca^2+^ homeostasis (Toescu et al., 2004).

In this study, by use of submerged hippocampal slices that allows simultaneous electrophysiological recordings and imaging, we were able to correlate directly neuronal network oscillations with mitochondrial activity. We show that the initiation of kainate-evoked gamma activity induces a mitochondrial depolarization response. Furthermore, mitochondrial depolarization, induced either chronically by the aging process or acutely by administration of mitochondrial protonophores, strongly inhibited gamma activity, indicating that the complex network activity required by the maintenance of gamma activity is susceptible to changes and modulations in mitochondrial status.

## 2 Methods

Male C57Bl/J6 mice (Ageing Colony of the University of Newcastle, UK), fed ad libitum and showed in previous studies to show age-dependent alteration in gamma activity (Vreugdenhil and Toescu, 2005) and impairment of spatial memory (Bach et al., 1999), were used either as young adults (2–5 months) or aged (22–25 months). Experiments conformed to the UK Animals (Scientific Procedures) Act 1986 and care was taken to minimize the number of animals and their suffering. Mice were anesthetized by medetomidine (1 mg/kg) and ketamine (76 mg/kg) intraperitoneal injection and killed by cervical dislocation. The brain was removed and chilled in cutting solution containing 189 mM sucrose, 2.5 mM KCl, 0.5 mM CaCl_2_, 10 mM MgCl_2_, 26 mM NaHCO_3_, 1.2 mM NaH_2_PO_4_, and 10 mM d-glucose at pH 7.4. Horizontal hippocampal slices (300 μm thick) were cut (Integraslice, Campden Instruments, Loughborough, UK) from the ventral hippocampus and placed in an interface-type storage chamber between artificial cerebrospinal fluid (aCSF) and moist carbogen at 23 °C, the aCSF contained 125 mM NaCl, 3 mM KCl, 2 mM CaCl_2_, 1 mM MgCl_2_, 26 mM NaHCO_3_, 1.25 mM NaH_2_PO_4_, 10 mM d-glucose; pH 7.4. After 1 hour of rest, the slices were transferred to submerged conditions, into a series 20 perfusion bath (Harvard Instruments, Edenbridge, UK) (300 μL per mm of solution height) onto the stage of an inverted microscope (Olympus BX50WI, Southend, UK) and perfused with aCSF at a rate of 7 mL/minute. To ensure perfusion on both sides of the slice (Hájos et al., 2009), the slice was placed on glass fibers (50 μm diameter), and glued in parallel with the flow to the bottom of the recording chamber. Temperature of the perfusion fluid was kept relatively low (29 °C) to minimize metabolic stress and to ensure the optimal conditions for the development of gamma activity in in vitro conditions.

### 2.1 Field potential recordings

Extracellular field potentials were recorded using aCSF-filled micropipettes (4–5 MΩ) placed in CA3c stratum pyramidale (stratum oriens side). Signals were amplified with Neurolog Nl104 AC-coupled amplifiers (Digitimer; Welwyn Garden City, UK), band-pass (2–500 Hz) Bessel filtered with a Neurolog NL-125 unit (Digitimer), digitized and sampled at 2 kHz using a 1401 plus A-D converter (Cambridge Electronic Design, Cambridge, UK). The incoming digital stream was analyzed using Spike-2 software (Cambridge Electronic Design). To counteract the possible noise from the 50 Hz mains, for all recordings we used a Humbug (Digitimer). The power of network oscillations was measured over 20 to 100 seconds of data recordings, using the fast Fourier transformation, which quantifies the proportional power of each wavelength within the section of data. Power spectra provide a quantitative measure of the frequency components of a recording and represents oscillatory strength in individual frequencies. To account for the temperature dependence of the oscillation frequency (Dickinson et al., 2003), the gamma frequency band was considered to be between 20 and 60 Hz. Data are expressed as summated power values, calculated as the sum of the power of the 1 Hz bins between 20 and 60 Hz.

#### 2.1.1 Assessing changes in mitochondrial membrane potential

Mitochondrial membrane potential (Ψ_m_) was measured in hippocampal slices using the fluorescent dye Rhodamine 123 (R123) that distributes in a Nerstian fashion in the cellular compartments according to their membrane potential (Toescu and Verkhratsky, 2000). At concentrations which do not inhibit significantly mitochondrial function, R123 is a sensitive and specific probe of Ψ_m_ that has been proved useful in measuring the changes in Ψ_m_ in various tissues including brain slices (Ward et al., 2000; Xiong et al., 2002). R123 solutions were freshly prepared daily and loaded under identical conditions for both young and aged slices. The slices were transferred to a smaller incubation chamber (2 mL volume), filled with 1 mL aCSF containing 10 μM R123 and left for 25 minutes at room temperature with the medium continually bubbled with 95% O_2_ and 5% CO_2_. After a wash, the slice was placed in a perfusion chamber on the stage of the microscope, and visualized through a 40× water-immersion objective. The intensifier gain on the camera was always set at the same value for consistency of fluorescent readings and allowing direct comparison between groups. Images were taken using an intensified GenIV camera (Roper Instruments, Marlow, UK), with a 12-bit digital output, with 4096 gray levels. The R123 excitation light (490 nm) was provided by a monochromator (Cairn Research, Ltd., Faversham, UK). The emission filters (550-nm cutoff for R123) were placed in a Sutter filter wheel installed in front of the camera. Both excitation and emission conditions were controlled by the software (MetaFluor, Molecular Devices Corp., Downington, PA, USA). Images were captured using the MetaFluor/MetaMorph software, version 7.0 (Molecular Devices Corp.), which provides also comprehensive image analysis. In slices and without confocal optical conditions, the identification of individual cells loaded with R123 is difficult, and thus, for image fluorescence analysis, we integrated the signal from a large region of interest (ROI) (512 × 512 pixels), using the 20× or 40× water immersion objectives. The images were centered on the stratum pyramidale of the CA3 region, and covered most of this region. We chose to record the R123 fluorescence images at this scale of magnification, rather than focusing at the level of individual neurons, as to be in keeping with the scale of the electrophysiological measurements that recorded field potentials (electrodes usually situated at the periphery of the field of view, see above for placement).

In control experiments, under resting conditions and in the absence of stimulation, the sampling of the R123 fluorescence over the recording region of interest showed no significant or systematic drifting over periods of up to 40 minutes.

#### 2.1.2 Calcium imaging

Our initial aim was to record intracellular Ca^2+^ ([Ca^2+^]_i_) signals from a large part of area CA3 by use of bulk fluorescent Ca^2+^ dye loading (fura-2 AM, Molecular Probes, Life Technologies, Paisley, UK) of the slices (Peterlin et al., 2000; Xiong et al., 2002), but this proved unsuccessful in the hippocampal aged slices. Eventually, the only reliable and consistent method of loading the aged neurons was the use of patch pipettes. Whole-cell patch clamp recordings were performed from neurons in CA3c stratum pyramidale by using 3- to 7-MΩ borosilicate pipettes filled with 135 mM potassium methylsulfonate, 15 mM KCl, 10 mM NaCl, 10 mM Hepes, 0.5 mM MgATP, and 0.05 mM fura-2 pentapotassium salt (Molecular Probes, Invitrogen). A GΩ seal was formed (measured by an Axopatch 2D amplifier, Axon Instruments, Foster City, CA), and whole-cell configuration achieved, with the access resistance < 20 MΩ.

Neuronal fluorescence increased over the soma to a maximum fluorescence value after 5–10 minutes. Fluorescence of fura-2-loaded CA3 neurons was imaged using a 40× water-immersion objective lens and an upright microscope (Olympus BX50WI). The excitation light 340 and 380 nm for fura-2 experiments was provided by a monochromator (Cairn Research, Ltd.) while the emission filter (510 nm) was placed in the Sutter filter wheel placed in front of the camera. Fluorescent images were taken using a cooled intensified GenIV camera (Roper Instruments) with a 12-bit digital output. The preparation was illuminated by alternating the 340-nm and 380-nm wavelengths, and pairs of fluorescence images were acquired every 5 seconds. The ratio images were calculated by dividing the 340-nm image by the corresponding 380-nm image, after subtraction of background fluorescence.

### 2.2 Materials and statistics

All chemicals were purchased from Sigma (Poole, UK), unless specified otherwise. Data are presented throughout as mean ± standard error of the mean (SEM). Statistical comparisons were made between experimental groups, using Student *t* test, with the resulting *p* values being presented.

## 3 Results

### 3.1 Gamma oscillations in submerged slices

**Fig. 1:**
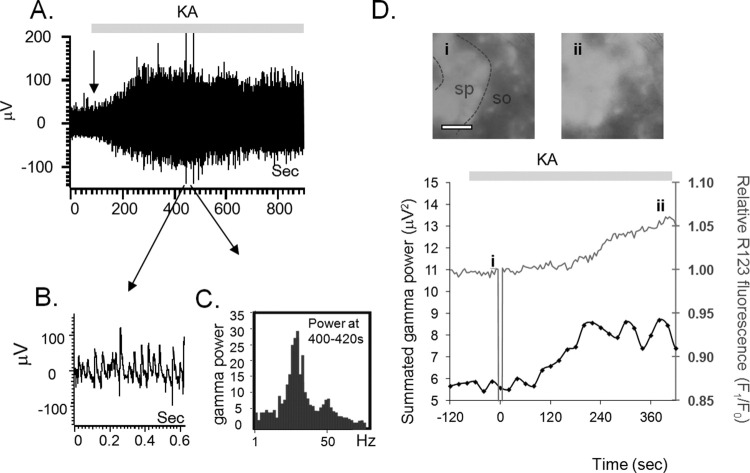
Example of combined recordings of gamma oscillations and mitochondrial depolarization status in a submerged hippocampal slice. Hippocampal slices were bath loaded with 1 mM Rhodamine 123 (R123) and then perfused with oxygenated artificial cerebrospinal fluid (aCSF). The fluorescence signal from the whole submerged slice was imaged through a 40× water immersion objective. Field potentials were simultaneously recorded from CA3 stratum pyramidale. (A) Field potential recordings in stratum pyramidale before and after application of 250 nM kainate; (B) raw electrophysiological activity on an expanded time course, and from consecutive 20-second bins, the power spectrum was calculated (C). (D) R123 fluorescence signal (gray upper trace) was averaged across the objective's field of view, and all values were normalized (as F_1_/F_0_ values, on the right-hand side ordinate axis) to the average value of R123 fluorescence in the 2 minutes preceding the application of kainate, indicated by the gray bar at the top of the traces. The fluorescence images before (i), and after (ii) kainate administration, are illustrated in the top insets (scale bar, 100 μm). The lower trace (black squares) in the panel shows the summated gamma power of the oscillations in each of the 20-second bins analyzed. Sp, stratum pyramidale; so, stratum oriens.

The main purpose of this study was to explore the extent of association between mitochondrial activity and gamma oscillatory activity, particularly in the context of neuronal aging, characterized by decreased mitochondrial function (Toescu and Verkhratsky, 2007; Toescu and Vreugdenhil, 2010). Recording mitochondrial activity requires imaging of slices under submerged conditions while the large majority of the previous experiments analyzing gamma brain oscillations were performed on slices maintained in interface conditions (e.g., Driver et al., 2007; Vreugdenhil and Toescu, 2005). Data presented in Fig. 1 show that, with a high perfusion rate in the bath (7 mL/minute), kainate-induced oscillations can be induced and maintained also in submerged conditions, while the mitochondrial activity can be monitored through the changes in R123 signals.

For correlation between the R123 traces and electrophysiological recordings, the latter (example in Fig. 1A) were binned in sections of 20 seconds (Fig. 1B). For each such bin, a power spectrum was obtained (Fig. 1C) and the power of the oscillatory activity in the 20–59 Hz range was calculated. In Fig. 1D, this summated gamma power for each 20-second bin is plotted against time, alongside the average R123 fluorescence in the field of view. For direct comparison of the R123 readings between different slices, the R123 fluorescence values were normalized to the average R123 signal in the period just preceding stimulation, and this normalized value is presented in the graph in Fig. 1D.

In these experiments we showed that the application of kainate at a concentration that is able to generate gamma oscillation activity, was followed, after a delay, by a mitochondrial depolarization response (cf., Fig. 1D). Both the delay of the mitochondrial depolarization response, and the time difference between the initiation of gamma oscillations (defined as the first data point, after the addition of kainate, that precedes a sequence of 3 consecutive increases in the summated gamma power) and the subsequent mitochondrial response were sensitive to the kainate concentration. An increase in kainate concentration from 100 to 250 nM accelerated the initiation of the mitochondrial depolarization response from 277 ± 34 seconds to 130 ± 10 seconds (Student *t* test *p* < 0.001) and reduced the time interval between initiation of the gamma oscillations and mitochondrial response from 93 ± 18 seconds with 100 nM kainate to 65 ± 10 seconds with 250 nM kainate, (Student *t* test *p* = 0.021).

### 3.2 Effect of aging

**Fig. 2:**
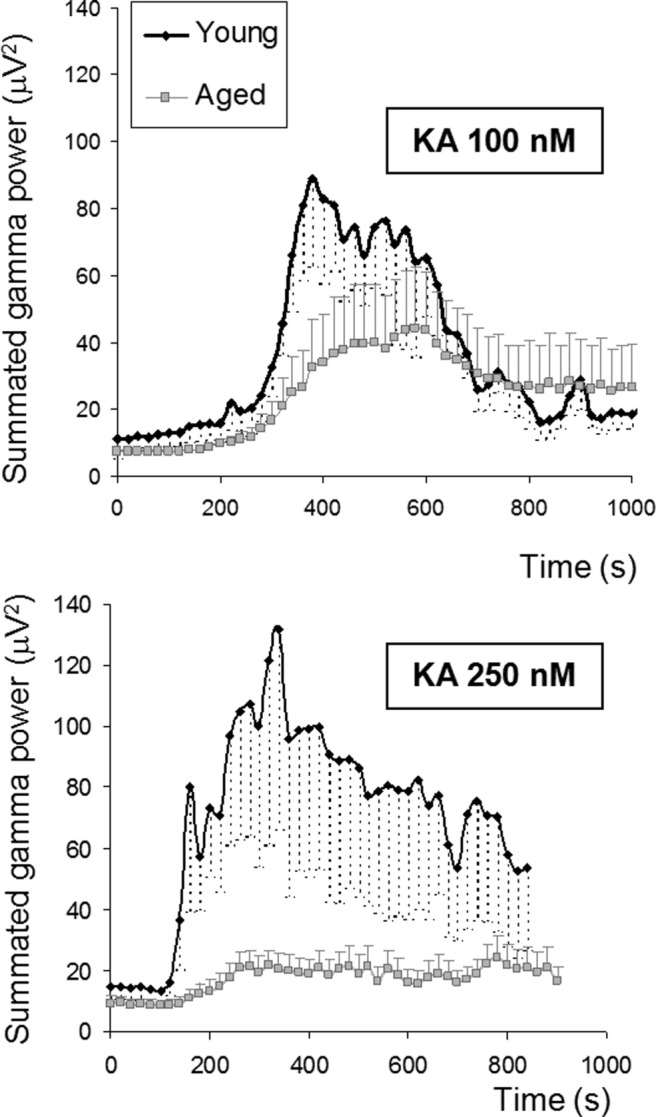
Effect of age on kainate-evoked gamma oscillations in submerged hippocampal slices. In the top panel gamma oscillation power (average power in 20–60 Hz) is shown as function of time after application of 100 nM kainate for 11 slices from young mice (black symbols) and 6 slices from aged mice (gray symbols). Data are mean ± standard error of the mean (SEM). Bottom panel shows the same as above, but for exposure to 250 nM kainate in 8 slices from young mice and 9 slices from aged mice.

We also show that the age-dependent reduction in the summated power of the activity in the gamma range previously reported (Vreugdenhil and Toescu, 2005) can also be demonstrated in submerged slices. As shown in Fig. 2, the maximal summated gamma power was significantly smaller in the aged slices, with a 70% decrease when the gamma oscillations were triggered by 100 nM kainite from 148.8 ± 41.7 μV^2^ in young adult (*n* = 11 slices; 6 young animals) to 43.8 ± 28.9 in the aged (*n* = 6 slices; 4 aged mice), Student (*t* test *p* = 0.03) and an 84% decrease for the 250 nM kainate (from 216.6 ± 65.9 μV^2^) for the young adult (*n* = 8, 5 animals) to 35.5 ± 12.1 for the aged animals (*n* = 9, 5 animals), Student *t* test *p* = 0.01. The time to peak, measured from the time of kainate application to the time of maximal peak of summated gamma power, while sensitive to the concentration of kainate used (e.g., for the young adult animals the time to maximum was 440 ± 43 seconds (100 nM kainate) versus 175 ± 22 seconds (250 nM kainate), Student *t* test *p* = 0.006, was not significantly affected by the age of slices. In agreement with our previous report (Vreugdenhil and Toescu, 2005), the age-associated changes in the power of the gamma oscillations were not associated with changes in peak frequency (35 ± 3 Hz vs. 27 ± 3, aged vs. young, respectively, for 100 nM kainate (KA); and 28 ± 2 and 29 ± 2, aged vs. young, respectively, for 250 nM KA protocols).

**Fig. 3:**
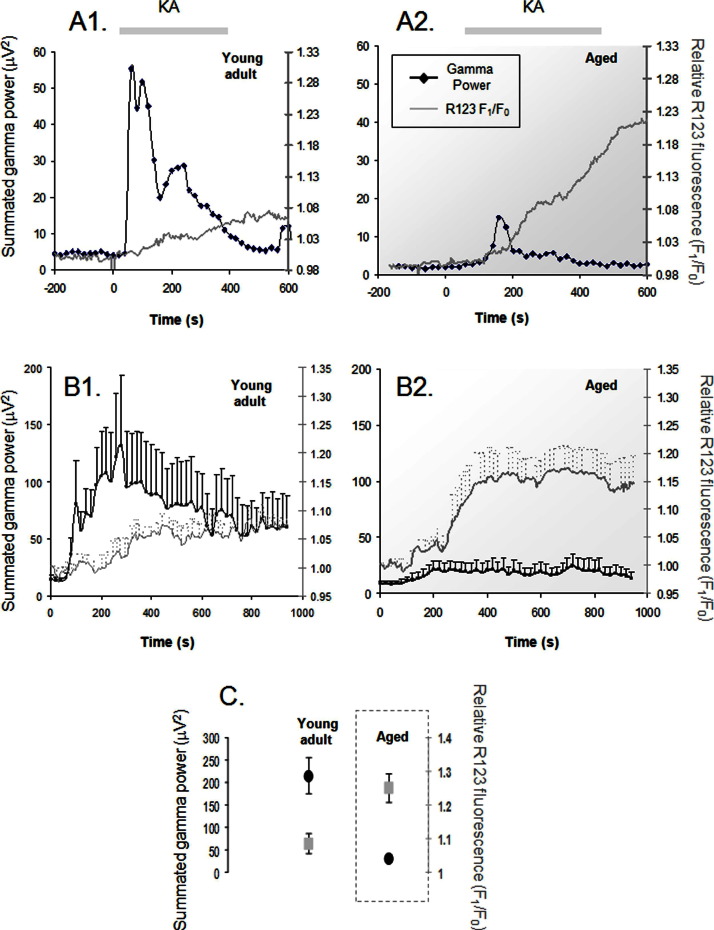
Effect of age on the gamma oscillations and on the associated mitochondrial membrane potential response induced by 250 nM kainate. (A) Examples of individual slice responses to kainate application; the rhodamine signal (right ordinate) is normalized to the pre-kainate period (see legend to Fig. 2), while the left ordinate shows the summated power of the activity in the range 20–60 Hz, calculated from the temporal bin of 20 seconds. While (A1) shows a presentative example of a recording from slices from young animals, with a large gamma power response and a small mitochondrial depolarization response, (A2) shows a typical response from aged slices: a much reduced gamma power with a large mitochondrial depolarization. (B) The average traces (mean ± standard error of the mean [SEM]) for both summated power and normalized Rhodamine 123 (R123) fluorescence for the young slices (B1, *n* = 8) and the aged slices (B2, *n* = 9). Note, for comparison purposes, that the summated power average traces in (B1) and (B2) are derived from the same set of data shown in Fig. 2, lower (250 nM) panel. (C) Average values (mean ± SEM) of the maximal gamma summated power and normalized R123 fluorescence increase for the 2 age groups.

Taking into account the known alterations of mitochondrial function with age (Beal, 2005; Boveris and Navarro, 2008; Mattson et al., 2008; Xiong et al., 2002), we next investigated the relationship between mitochondrial depolarization and gamma oscillation power as a function of age. Fig. 3A shows typical examples of the simultaneous development of gamma oscillation power and mitochondrial depolarization in response to 250 nM kainate in a slice from a young mouse (Fig. 3A1) and, plotted on the same scale, the responses recorded in a slice from an aged mouse (Fig. 3A2). The age-related decrease in gamma oscillation power was typically associated with a larger mitochondrial depolarization response, and this is illustrated by the average response to kainate in Fig. 3B1, for 8 young adult slices (5 animals), and in Fig. 3B2, for 9 aged slices (5 animals). In comparison with the relatively slow and small R123 signal (a ratio of R123 fluorescence, before and after the admission of kainite, of 1.08 ± 0.03) that accompany the relatively strong kainate-evoked gamma oscillation in the young slices, there is, in the aged slices, a 300% increase in the mitochondrial depolarization (1.25 ± 0.04 in the aged animals, Student *t* test *p* = 0.001) that accompany the relatively weak gamma oscillation (Fig. 3C).

### 3.3 Effect of mitochondrial depolarization on gamma oscillations

**Fig. 4:**
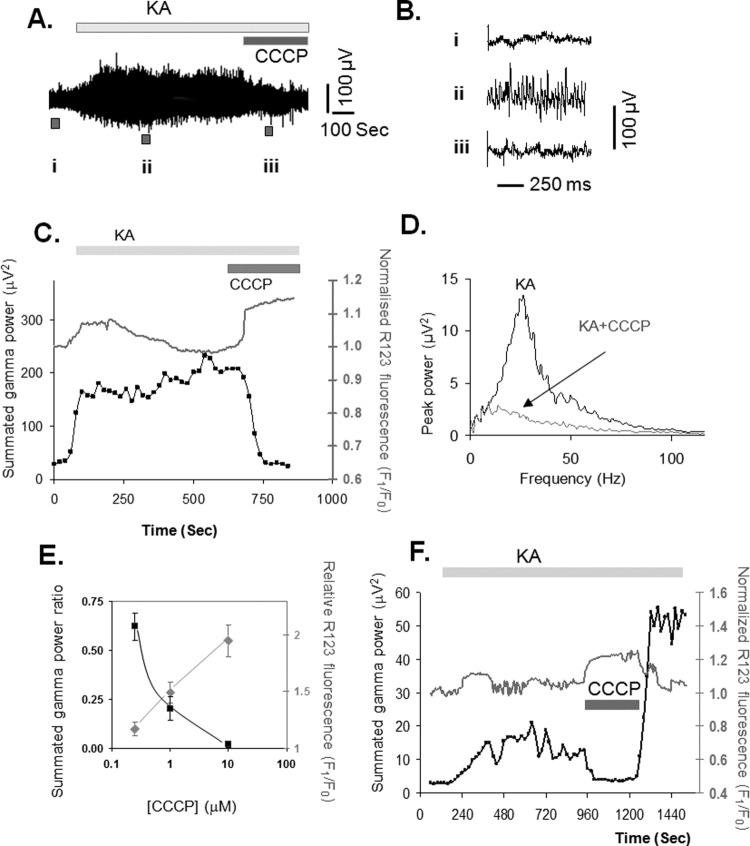
Effect of a mitochondrial uncoupler on the mitochondrial membrane potential and gamma oscillations elicited by 250 nM kainate in a young slice. After the establishment of gamma oscillations, a mitochondrial protonophore, carbonylcyanide m-chlorophenylhydrazone (CCCP, 1 μM) was added to the perfusion circuit. (A) A field potential recording from CA3 stratum pyramidale of a young slice, while the traces in (B) show, on an expanded time scale, the characteristics of the electrical activity at the various time points during the protocol as marked on the main trace. (C) The relationship between the Rhodamine 123 (R123) measurements and the summated gamma power during kainate-evoked activity and following the application of 1 μM CCCP. (D) The effect of the protonophore on the power spectrum of the kainate (250 nM)-evoked activity. (E) The inverse relationship between the increased mitochondrial depolarization as measured by the increase in R123 fluorescence and the summated gamma power, expressed as a ratio between the summated power after and before the application of the respective CCCP concentrations (each data point shows mean ± standard error of the mean [SEM] from 3 to 5 young slices). (F) Example of a slice in which the removal of CCCP (0.25 μM) was associated with a significant increase in summated gamma power.

The data presented in Fig. 3 suggest an inverse relationship between mitochondrial depolarization and gamma power, such that an increased mitochondrial depolarization is associated with a depressed gamma oscillation power. Because in the aged brain the mitochondria are chronically depolarized (Hagen et al., 1997; Xiong et al., 2002), we wanted to separate the specific effect of mitochondrial depolarization from that of age. To test for these effects, we have used, in young adult slices, a mitochondrial uncoupler, carbonylcyanide m-chlorophenylhydrazone (CCCP), that acts as a direct depolarizing agent by allowing proton flux across the inner mitochondrial membrane. At large concentrations (10 μM), CCCP strongly depolarized the mitochondria, as reflected by a large increase in the R123 fluorescence signal (the normalized R123 fluorescence ratio increased to 2.1 ± 0.1, *n* = 5; cf. Fig. 4E), and caused, within 5–10 minutes, morphological changes indicative of severe metabolic impairment, such as large increases in cell volume in bright field images and sudden and large fluorescence losses (data not shown). At lower concentrations (1 μM), CCCP applied during the kainate-evoked oscillatory activity (Fig. 4A and C), induced a small increase in R123 fluorescence (Fig. 4C) that was immediately followed by a dramatic reduction in the gamma power (Fig. 4A, B, and C). Analysis of the power spectra (Fig. 4D) showed that CCCP suppressed the kainate-evoked oscillation power especially in the gamma band. The graph in Fig. 4E confirms this inverse correlation across a range of CCCP concentrations.

Importantly, the use of R123 as a mitochondrial membrane potential dye with limited membrane permeability (Ward et al., 2000), allows its use as an indirect means of monitoring the recovery of the mitochondrial membrane potential (Toescu and Verkhratsky, 2000). At concentrations as low as 0.25 μM, CCCP was still able to induce a small, detectable mitochondrial depolarization (Fig. 4E and F), which was sufficient to suppress the gamma oscillations. When the protonophore was washed out, the mitochondrial membrane potential recovered and the gamma oscillation was re-established (Fig. 4F).

### 3.4 Kainate-evoked [Ca^2+^]_i_ signals

**Fig. 5:**
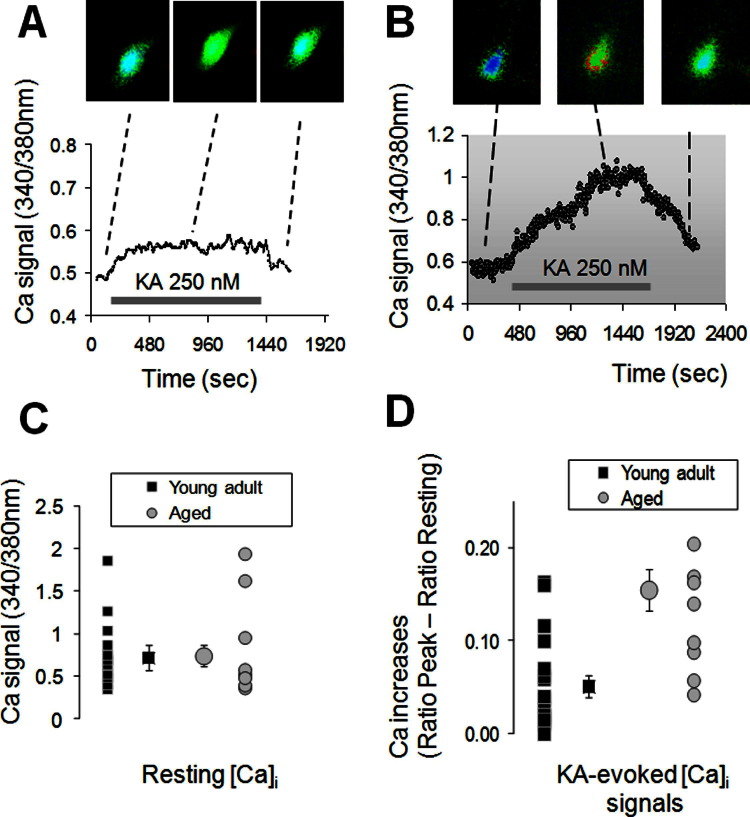
Effect of age on the kainate-evoked Ca signals in individual CA3 neurons. (A) Example of the intracellular Ca^2+^ ([Ca^2+^]_i_) response (ratio between the fura-2 fluorescence at 340 and 380 nm) recorded from a CA3 cell in a slice from a young mouse, in response to 250 nM kainate. (B) Example of the [Ca^2+^]_i_ response as in (A) for a cell from a slice of an aged mouse. (C) Scatterplot of the resting [Ca^2+^]_i_ values for the 2 age groups, with the larger central symbol showing the mean (± standard error of the mean [SEM]) values. In a similar manner, (D) shows the individual values and the respective mean value for the maximal [Ca^2+^]_i_ evoked by 250 nM kainate in the 2 age groups. The [Ca^2+^]_i_ values are represented here as the difference, in ratio units, between the peak and the resting [Ca^2+^]_i_ values. Squares represent young, and circles, aged).

The initial kainate-evoked mitochondrial depolarization response is mainly determined by the cytosolic Ca^2+^ signal (Shuttleworth et al., 2003), that is initiated by the activation of either metabotropic or ionotropic kainate receptors (Lerma, 2006). We thus measured the kainate-evoked Ca^2+^ signal evoked in individual neurons patched in area CA3 stratum pyramidale. As shown in Fig. 5, and as reported before for either area CA1 (Thibault et al., 2001) or cerebellum (Xiong et al., 2002), the values of the resting [Ca^2+^]_i_ were not different between young adult and aged CA3 neurons (fura-2 ratio of 0.78 ± 0.14, for the young adult; *n* = 12; 4 animals vs. 0.86 ± 0.21, for the aged; *n* = 8; 5 animals; Fig. 5C).

Application of 250 nM kainate induced a [Ca^2+^]_i_ signal in both young adult and aged neurons, as shown in Fig. 5A and B. The [Ca^2+^]_i_ signal in the young adult slices was significantly quicker than in the aged, as reflected in the time-to-peak (154 ± 39 seconds, young adult; *n* = 7;4 animals vs. 322 ± 81 seconds, aged; *n* = 7; 5 animals; Student *t* test *p* = 0.032). In addition to this delay, Fig. 5D indicates that the aged neurons also showed a significant increase in the amplitude of the kainate-evoked [Ca^2+^]_i_ signals—the maximal changes in the [Ca^2+^]_i_, as estimated from the differences in fura-2 ratio between response and resting, were 0.051 ± 0.01 in the young adult neurons versus 0.155 ± 0.02 the aged neurons (Student *t* test *p* < 0.001). In agreement with a previous report (Lu et al., 2011a), in the absence of the KA stimulation there was no age-related difference in the basic electrophysiological properties (resting membrane potential, membrane resistance and membrane capacitance, data not shown) of the studied neurons; whereas admission of KA evoked a larger depolarization response in the aged neurons than in the young (3.9 ± 1.1 mV vs. 11.1 ± 1.3 mV in aged; *p* < 0.001).

## 4 Discussion

Our results show that the initiation of the kainate-induced gamma oscillation activity induced an increased R123 fluorescence signal that is a functional marker for mitochondrial depolarization (Xiong et al., 2002, 2004) resulting from increased levels of metabolic activity. Earlier measurements of mitochondrial activity in the hippocampal region showed mitochondrial depolarization in response to stimulus train-induced bursting activity that correlated well, both temporally and spatially, with the level of intensity of stimulation (Bindokas et al., 1999), and were larger in CA3 than in CA1 (Bindokas et al., 1999; Kann et al., 2011). Similar regional differences were observed in the response to glutamate iontophoresis or bath application of kainate (Shuttleworth et al., 2003). To correlate with the population nature of the field recordings that measured oscillatory activity, our R123 fluorescence measurements were recorded from the whole field of view, rather than from individual cells. In such conditions, the actual cellular source of the mitochondrial signal recorded was not identified, but it is important to note that during gamma oscillations, the interneurons, that represent only less than 20% of the cells in the hippocampus (Caeser and Aertsen, 1991), are firing at rates of up to 40 Hz, whereas the pyramidal neurons generate action potentials only at 1–3 Hz (Hájos et al., 2004). These high rates of neuronal activity probably explain why gamma oscillations are driving the mitochondrial oxidative phosphorylation at almost maximal capacity (Kann et al., 2011). At the subcellular level, mitochondrial redox responses to gamma oscillations appear larger in the synaptic compartment, as indicated by the fact that the increases in NAD(P)H (a reduced form of NAD(P): nicotinamide adenine dinucleotide (phosphate)) fluorescence, reflecting increased mitochondrial metabolism, were reportedly larger in stratum radiatum of area CA3 than in stratum pyramidale (Huchzermeyer et al., 2008), and larger in the CA3 region than in CA1 (Kann et al., 2011). However, a significant level of functional heterogeneity has been detected at the level of individual mitochondria from different compartments of the neurons (Young et al., 2008), together with a possible shift of levels of activity between dendritic and somatic compartments, as a function of the level of stimulation (Kovács et al., 2005). In addition, it is important to note that measurements at a regional level will also include, per force, signals generated in other neural cells, such as glia. Although it is well established that there is an important neuronal-glia metabolic crosstalk (Attwell et al., 2010; Magistretti, 2006), only very few studies have investigated the participation of glia in gamma activity (Sakatani et al., 2008)or the effects of aging on glial mitochondrial activity (Boumezbeur et al., 2010).

An important mechanism of mitochondrial depolarization is the uptake of cytosolic Ca^2+^ (Brustovetsky and Dubinsky, 2000). Many previous studies have shown that rises in [Ca^2+^]_i_ induce mitochondrial depolarization, either in acutely dissociated (Duchen, 1992) or in cultured neurons (Xiong et al., 2004). This depolarization results from mitochondrial Ca^2+^ uptake (Nowicky and Duchen, 1998), through an electrogenic Ca^2+^ uniporter present on the inner mitochondrial membrane, which is a Ca^2+^-selective ion channel, with a strong inward rectification (Kirichok et al., 2004), recently identified as a 40 kDa protein on the inner mitochondrial membrane with 2 transmembrane domains able to generate a pore-forming region (De Stefani et al., 2011). The uptake process is activated by Ca^2+^ (Gunter et al., 1998; Moreau et al., 2006), shows a Ca^2+^ concentration threshold (Xiong et al., 2004; Young et al., 2008), and, at least in some cell types, has an incomplete, slow Ca^2+^-dependent inactivation (Moreau et al., 2006).

The involvement of kainate receptors in gamma oscillations is well established (Fisahn, 2005; Fisahn et al., 2004) and it is becoming increasingly clear that these receptors, as heteromers generated from 5 distinct subunits (Lerma, 2006), can exert both iono- and metabotropic effects, sometimes by activating different subunits at the same location (Ruiz et al., 2005). In response to stimulation with neurotoxic (> 50 μM) or epileptogenic (> 1 μM) concentrations of kainate, previous studies have reported both mitochondrial depolarization (Rego et al., 2001) and mitochondrial Ca^2+^ accumulation (Hoyt et al., 1998; Peng and Greenamyre, 1998; Rego et al., 2001), at the same time with increases in oxygen consumption (Kunz et al., 1999). Here we show that kainate, even at nanomolar concentrations, is able to induce both a Ca^2+^ signal and a mitochondrial depolarization response. The source for the Ca^2+^ signal generated by kainate was not investigated further in this study; but could be a combination of activation of voltage-operated Ca^2+^ channels (VOCCs) following the kainate-evoked neuronal depolarization (Fisahn et al., 2004; Lu et al., 2011a), or Ca^2+^ entry through the kainate receptors (Egebjerg and Heinemann, 1993), or a release of Ca^2+^ from the intracellular stores, through an IP_3_-mediated process (Mathew and Hablitz, 2008). In the aged slices, we found that kainate evokes a larger Ca^2+^ signal (Fig. 5) and it is important to note that either of the mechanisms described above would be consistent with this observation because both an increase in the activity of the voltage-operated Ca^2+^ channels and a sensitization of the intracellular Ca^2+^ stores has been described in the aged hippocampal neurons (Thibault et al., 2007). Another possible source for the increased [Ca^2+^]_i_ signal in the aged neurons is the increase in the threshold for the mitochondrial Ca^2+^ uptake, that results from the chronic mitochondrial depolarization in the aged neurons, as demonstrated in the cerebellar granule neurons (Xiong et al., 2004).

One striking observation in the present study is the opposite effects of aging: while depressing the gamma oscillation (Fig. 2, and Driver et al., 2007; Vreugdenhil and Toescu, 2005), aging increases significantly the mitochondrial depolarization response (cf., Fig. 3). This inverse relationship between gamma oscillations and mitochondrial depolarization was also observed in conditions in which mitochondria were directly depolarized by low, nontoxic concentrations of mitochondrial protonophores (cf.. Figs. 3 and 4), and also during the spontaneous development of gamma oscillations (cf., Fig. 1). That the effects of protonophores are similar to those of aging is not that surprising, because the mitochondria are chronically depolarized in the aged neurons from various parts of the brain (Hagen et al., 1997; Parihar and Brewer, 2007; Xiong et al., 2002; but see LaFrance et al., 2005). This disturbance in mitochondrial performance has been attributed mainly to the age-induced, and free radical-mediated, effects in the activity of various complexes of the respiratory chain. In the hippocampus, Complex I and IV show decreased activity, whereas Complexes II and III are relatively spared (Navarro and Boveris, 2007; Navarro et al., 2008). Because in addition to the Ca^2+^ uptake-induced depolarization, other processes such as metabolic substrate availability, presence, and generation of free radicals, changes in ATP/ADP (adenosine triphosphate/adenosine diphosphate) ratio as well as flickering opening of the mitochondrial permeability transition pore will affect the value of the mitochondrial membrane potential (Toescu, 2000), it is likely that defects in the efficiency of the electron transport complexes could also be a contributing factor for the increased mitochondrial depolarization response that follows neuronal activation in aged neurons, as shown here and in previous studies (Parihar and Brewer, 2007; Vergun et al., 1999).

On their own, in the aged brain, such mitochondrial dysfunction is not sufficient to cause neuronal death, but only to decrease the homeostatic reserve (Toescu, 2005), resulting in functional impairment in special conditions, such as higher energetic demands (Kann et al., 2011; Lu et al., 2011a) or following reductions in tissue perfusion, as following ischemia (Barth and Mody, 2011). The present set of data indicate that similar functional limitations can affect complex network activities, such as gamma oscillations, that facilitates effective communication of various neuronal groups with tight temporal and spatial integration parameters. Indeed, by use of NAD(P)H measurements as indicators of overall mitochondrial redox efficiency, it was shown that gamma oscillations are very sensitive to small reductions in mitochondrial efficiency (Huchzermeyer et al., 2008) or to the specific inhibition of Complex I activity by rotenone (Kann et al., 2011). Importantly, such inhibition of gamma oscillations appeared at stages where interneuronal synaptic activity and neuronal population responses were not affected (Huchzermeyer et al., 2008).

Gamma oscillations provide the millisecond-precision time frame for neuronal discharges and, because it also facilitates the effective synchronization of inputs at various spatial locale (i.e., spatial summation of excitatory postsynaptic potentials, EPSPs), it enhances the probability of coinciding pre- and postsynaptic discharges, required for Hebbian plasticity. With aging, the LTP/LTD (long term potentiation/long term depression) balance is in favor of LTD (Rosenzweig and Barnes, 2003), which further emphasizes the need for effective integration to achieve coincident firing. In addition, during the gamma cycle, stimulus intensity is translated into firing time in respect to gamma phase (Fries et al., 2007). Impairing this ability would fit well with the decreased signal-to-noise ratio reported for aged brains, characterized by increased spontaneous/nonspecific firing (Turner et al., 2005; Yang et al., 2009) and decreased synchronization (McEchron et al., 2001). The metabolic limitations for gamma oscillations reported here for the aged hippocampus are unlikely restricted to the hippocampus and may well contribute to the mild cognitive impairment seen in normal brain aging.

## Disclosure statement

The authors disclose no conflicts of interest.

Experiments conformed to the UK Animals (Scientific Procedures) Act 1986 and care was taken to minimize the number of animals and their suffering.
